# Educational attainment as a modifier for the effect of polygenic scores for cardiovascular risk factors: cross-sectional and prospective analysis of UK Biobank

**DOI:** 10.1093/ije/dyac002

**Published:** 2022-02-03

**Authors:** Alice R Carter, Sean Harrison, Dipender Gill, George Davey Smith, Amy E Taylor, Laura D Howe, Neil M Davies

**Affiliations:** MRC Integrative Epidemiology Unit, University of Bristol Bristol, UK; Population Health Sciences, Bristol Medical School, University of Bristol, Bristol, UK; MRC Integrative Epidemiology Unit, University of Bristol Bristol, UK; Population Health Sciences, Bristol Medical School, University of Bristol, Bristol, UK; Clinical Pharmacology and Therapeutics Section, Institute of Medical and Biomedical Education and Institute for Infection and Immunity, St George’s, University of London, London, UK; Clinical Pharmacology Group, Pharmacy and Medicines Directorate, St George’s University Hospitals NHS Foundation Trust, London, UK; Novo Nordisk Research Centre Oxford, Old Road Campus, Oxford, UK; Department of Epidemiology and Biostatistics, School of Public Health, Imperial College London, London, UK; MRC Integrative Epidemiology Unit, University of Bristol Bristol, UK; Population Health Sciences, Bristol Medical School, University of Bristol, Bristol, UK; NIHR Bristol Biomedical Research Centre, University of Bristol, Bristol, UK; MRC Integrative Epidemiology Unit, University of Bristol Bristol, UK; Population Health Sciences, Bristol Medical School, University of Bristol, Bristol, UK; NIHR Bristol Biomedical Research Centre, University of Bristol, Bristol, UK; MRC Integrative Epidemiology Unit, University of Bristol Bristol, UK; Population Health Sciences, Bristol Medical School, University of Bristol, Bristol, UK; MRC Integrative Epidemiology Unit, University of Bristol Bristol, UK; Population Health Sciences, Bristol Medical School, University of Bristol, Bristol, UK; K.G. Jebsen Center for Genetic Epidemiology, Department of Public Health and Nursing, NTNU, Norwegian University of Science and Technology, Trondheim, Norway

**Keywords:** Polygenic scores, education, inequalities, cardiovascular disease, gene*environment interactions

## Abstract

**Background:**

Understanding the interplay between educational attainment and genetic predictors of cardiovascular risk may improve our understanding of the aetiology of educational inequalities in cardiovascular disease.

**Methods:**

In up to 320 120 UK Biobank participants of White British ancestry (mean age = 57 years, female 54%), we created polygenic scores for nine cardiovascular risk factors or diseases: alcohol consumption, body mass index, low-density lipoprotein cholesterol, lifetime smoking behaviour, systolic blood pressure, atrial fibrillation, coronary heart disease, type 2 diabetes and stroke. We estimated whether educational attainment modified genetic susceptibility to these risk factors and diseases.

**Results:**

On the additive scale, higher educational attainment reduced genetic susceptibility to higher body mass index, smoking, atrial fibrillation and type 2 diabetes, but increased genetic susceptibility to higher LDL-C and higher systolic blood pressure. On the multiplicative scale, there was evidence that higher educational attainment increased genetic susceptibility to atrial fibrillation and coronary heart disease, but little evidence of effect modification was found for all other traits considered.

**Conclusions:**

Educational attainment modifies the genetic susceptibility to some cardiovascular risk factors and diseases. The direction of this effect was mixed across traits considered and differences in associations between the effect of the polygenic score across strata of educational attainment was uniformly small. Therefore, any effect modification by education of genetic susceptibility to cardiovascular risk factors or diseases is unlikely to substantially explain the development of inequalities in cardiovascular risk.

Key Messages

- The role of educational attainment in modifying the effect of polygenic scores for a wide range of cardiovascular risk factors or diseases has not previously been studied.
- We explore whether educational attainment modifies the effects of polygenic susceptibility to alcohol consumption, body mass index, low-density lipoprotein cholesterol, lifetime smoking behaviour, systolic blood pressure, atrial fibrillation, coronary heart disease, type 2 diabetes and stroke.
- Effect modification by education was observed for some polygenic scores for cardiovascular risk factors, but not all.
- Effects were not always in the hypothesized direction and were dependent on the scale of analysis.
- Modification of the effect of genetic susceptibility to cardiovascular risk factors or cardiovascular disease by educational attainment is unlikely to substantially explain the development of inequalities in cardiovascular risk.

## Introduction

Socioeconomically deprived individuals have a greater risk of cardiovascular disease (CVD) than less deprived individuals.^1^ Most cardiovascular outcomes are multifactorial diseases with environmental and genetic aetiology.^2–4^ Therefore, it is plausible that socioeconomic position (SEP) may interact with, or modify, genetic susceptibility to CVD.

Large genome-wide association studies (GWASs) have identified many genetic variants associated with liability to CVD and its risk factors.^5–7^ Polygenic scores (PGSs) can subsequently be constructed, explaining substantial fractions of variation. Using UK Biobank, two studies have demonstrated that individuals with a higher Townsend deprivation index score have an accentuated risk of obesity in genetically susceptible adults.^8^^,^^9^ However, previous studies in the UK and Finland did not find evidence that education modified the effect of genetic susceptibility to high body mass index (BMI) on measured BMI.^9^^,^^10^

Whilst educational attainment, a measure of SEP, has been shown to modify the association of cardiovascular risk factors on CVD,^1^^,^^11^ it is unclear whether educational attainment modifies the effect of genetic susceptibility to a wide range of cardiovascular risk factors. If higher levels of education mitigate some of the genetic risk of cardiovascular risk (‘gene*environment interaction’), this may contribute to educational inequalities in CVD.^12^

Where two variables are known risk factors for an outcome, evidence of effect modification is expected on both, or one of, the additive or the multiplicative scale.^13^ Therefore, we carry out analyses on both scales. Identifying the magnitude and direction of any effect modification is of greatest importance for public health and in understanding the aetiology of cardiovascular inequalities.

## Methods

### UK Biobank

UK Biobank recruited 503 317 adults from the UK between 2006 and 2010, aged 37–73 years.^14^ Participants attended baseline assessment centres involving questionnaires, interviews and anthropometric, physical and genetic measurements.^14^^,^^15^ We use ≤320 120 individuals of White British ancestry (Supplementary Figure S1, available as Supplementary data at *IJE* online).

### Educational attainment

Participants reported their highest qualification achieved, which was converted to the International Standard Classification for Education (ISCED) coding for years of education (Supplementary Table S1, available as Supplementary data at *IJE* online).^16^ This definition has been used previously,^17^ including in UK Biobank.^18^

### Cardiovascular risk factors and cardiovascular disease

Cardiovascular risk factors were included if there was evidence for them being a causal risk factor for CVD from randomized–controlled trials, Mendelian randomization studies or clinical studies (see Supplementary Table S2, available as Supplementary data at *IJE* online) with suitable GWAS summary statistics available. Additionally, we included PGSs for several CVD outcomes. In total, nine PGSs were included in analyses: alcohol consumption,^19^ BMI,^20^ type 2 diabetes,^21^ low-density lipoprotein cholesterol (LDL-C),^22^ lifetime smoking behaviour,^23^ systolic blood pressure,^18^ atrial fibrillation,^24^ coronary heart disease (CHD)^6^ and stroke.^7^ Cardiovascular risk factors were measured at baseline, whilst incident cardiovascular outcomes (atrial fibrillation, CHD, stroke and type 2 diabetes) were determined prospectively by linked mortality records and hospital inpatient records (see Supplementary Table S3, available as Supplementary data at *IJE* online). A full description of how each risk factor/outcome was measured phenotypically and genetically is presented in the Supplementary Methods (available as Supplementary data at *IJE* online).

### Deriving polygenic scores

Summary statistics of the associations of the single-nucleotide polymorphisms (SNPs) with each cardiovascular risk factor/outcome were downloaded from MR-Base^25^ or directly from the relevant GWAS. Where possible, we used the most recent GWAS for each risk factor/outcome excluding UK Biobank participants to avoid sample overlap (See Table 1) (all GWAS were independent of UK Biobank with the exception of atrial fibrillation).

**Table 1:** Summary characteristics for each GWAS used to derive external weights in polygenic scores

Phenotype	Author/consortium	Population	Sample size (cases)	Unit
Alcohol consumption	GWAS and Sequencing Consortium of Alcohol and Nicotine Use^19^	European ancestry (summary statistics excluding UK Biobank)	630 154	Drinks per week
Body mass index	Genetic Investigation of Anthropometric Traits^20^	European ancestry	339 224	SD (kg/m^2^)
Low-density lipoprotein cholesterol	Global Lipids Genetics consortium^22^	European ancestry	188 578	SD (circulating lipids)
Smoking	Wootton *et al.*^23^	White British (split sample GWAS of UK Biobank; see Supplementary Methods, available as Supplementary data at *IJE* online)	318 147	SD (lifetime smoking index)
Systolic blood pressure	Carter *et al.*^18^	White British (split sample GWAS of UK Biobank; see Supplementary Methods, available as Supplementary data at *IJE* online)	318 147	SD (mm/Hg)
Atrial fibrillation	Roselli *et al.*^24^	Predominantly European (84.2%)	588 190 (65 446)	Log odds ratio
Coronary heart disease	CARDIoGRAMplusC4D^6^	Predominantly European (77%)	184 305 (60 801)	Log odds ratio
Type 2 diabetes	DIAbetes Genetics Replication And Meta-analysis^21^	European ancestry	159 208 (26 276)	Log odds ratio
Stroke	MEGASTROKE^7^	Predominantly European (85%)	521 612 (67 162)	Log odds ratio

SD, standard deviation; GWAS, genome-wide association study.

The 1000 Genomes Project was used to find proxy SNPs in linkage disequilibrium (LD) with SNPs not found in UK Biobank. Pruning of SNPs was carried out using the clump command in PLINK using an *r*^2^ parameter of 0.25 and a physical-distance threshold for clumping of 500 kB. PGSs were constructed using a range of *P*-value thresholds: *P* ≤ 5 × 10^–8^ (genome-wide significant), ≤0.05 and ≤0.5. As the *P*-value threshold increases, the variance explained by the PGS typically increases. However, increasing the numbers of SNPs increases the risk of pleiotropy and false-positive effects. Pruned SNPs from each GWAS were harmonized with SNPs from UK Biobank, aligning the effect estimates and alleles. Any SNPs that could not be harmonized, palindromic SNPs (where alleles on the forward and reverse strand are read the same) or triallelic SNPs were excluded. PGSs were created by multiplying the number of effect alleles for each participant by the association of the SNP with the phenotype in the GWAS, then summed across all SNPs for each phenotype. PGSs were standardized for use in analyses and reflect a 1 SD change.

Main analyses are presented using PGSs at the genome-wide significance threshold with other thresholds presented in the supplement.

### Exclusion criteria

Reverse causality can introduce bias when the temporality of the exposure and outcome is mis-specified and the outcome itself affects the exposure.^26^ Although CVD in adulthood cannot alter genetic variants determined at conception, and indeed is unlikely to change educational attainment typically determined in early adulthood, a diagnosis may lead to behavioural or lifestyle changes that change the relative importance of the PGS in determining the outcome. Participants were therefore excluded if they had experienced at least one diagnosis of any of the outcomes considered before baseline (atrial fibrillation, CHD, stroke and type 2 diabetes) or any one of myocardial infarction, angina, transient ischaemic attack, peripheral arterial disease, familial hypercholesterolaemia, type 1 diabetes and chronic kidney disease. These diagnoses can all result in statins being prescribed to prevent CVD, which may lead to behaviour change and therefore reverse causality.^27^ Diagnoses were ascertained through linked mortality data and hospital inpatient records using ICD-9 and ICD-10 codes (Supplementary Table S4, available as Supplementary data at *IJE* online).

Quality control of the genetic data was carried out using the Medical Research Council Integrative Epidemiology Unit quality-control pipeline, described in full previously.^28^ In brief, individuals were excluded if their genetic sex differed to their gender reported at baseline or for having aneuploidy of their sex chromosomes (non-XX or -XY chromosomes). Further individuals were excluded for extreme heterozygosity or a substantial proportion of missing genetic data. Related individuals were excluded, removing those related to the greatest number of other participants until no related pairs were left.^28^ This exclusion list was derived in‐house using an algorithm applied to the list of all the related pairs provided by UK Biobank (third-degree or closer) (Supplementary Figure S1, available as Supplementary data at *IJE* online). Individuals were excluded if they had withdrawn from UK Biobank or were, or may be, pregnant at baseline.

Individuals were further excluded if they were missing data for education, age and sex. Individuals were excluded from specific analyses if they were missing phenotypic measurements of the risk factor/outcome under consideration (see Supplementary Figure S1, available as Supplementary data at *IJE* online).

### Statistical analysis

#### Association of educational attainment with outcomes

Multivariable linear regression (adjusting for age and sex) was carried out to estimate the association between educational attainment and cardiovascular risk factors/outcomes.

#### Association between each polygenic score and observed phenotype

For each cardiovascular risk factor/outcome, we estimated the association between each PGS and the phenotype using multivariable regression, adjusting for age, sex and 40 genetic principal components to control for population structure. For continuous risk factors, measures were standardized, so estimates reflect the mean difference in SD of the phenotype, or natural log of the phenotype, per 1 SD higher PGS. For binary outcomes, estimates reflect the risk difference or odds ratio of the outcome per 1 SD higher PGS.

#### Effect modification by educational attainment on polygenic scores for cardiovascular risk

To test for effect modification, the linear model was stratified by years of educational attainment. To estimate the magnitude and direction of the effect modification, an interaction term was included in the linear model [e.g. PGS*education (continuous)]. Analyses were adjusted for age, sex and 40 genetic principal components. As effect modification is scale-dependent, tests of effect modification were carried out on both the additive and multiplicative scales.^13^ Additive and multiplicative effects were carried out as previously defined.^13^

### Secondary analyses

All analyses were replicated for PGSs at *P*-value thresholds of ≤0.05 and ≤0.5.

## Results

### UK Biobank cohort

Eligible UK Biobank participants (55% female) had a mean age of 57 (SD = 8.00) years. A higher proportion of participants (33%) left school after 20 years (equivalent to obtaining a degree) compared with those who left school after 7 years (equivalent to no formal qualifications) (16%) (Table 2).

**Table 2:** Descriptive characteristics of the main analysis sample compared with all individuals in UK Biobank at baseline who have not since withdrawn from the study

Variable	Analysis sample			Full UK Biobank	
	(*N* = 320 120)			(*N* = 502 156)	
Continuous variables	*N*		Mean (SD)	*N*	Mean (SD)
Age	320 120		56.66 (8.00)	502 156	56.54 (8.09)
Drinks per week	318 300		8.17 (9.05)	497 917	7.79 (9.05)
Body mass index	319 201		27.3 (4.72)	499 065	27.43 (4.8)
Low-density lipoprotein cholesterol	304 700		3.61 (0.86)	468 390	3.56 (0.87)
Systolic blood pressure	292 277		138.16 (18.58)	456 647	137.79 (18.62)
Smoking (lifetime behaviour)	301 684		0.32 (0.66)	318 112	0.34 (0.67)
Categorical variables	*N*		Frequency (%)	*N*	Frequency (%)
Sex	Female	320 120	175 108 (55)	502 156	273 025 (54)
Years of education	7 years	320 120	52 012 (16)	493 033	84 648 (17)
	10 years		54 899 (17)		82 357 (17)
	13 years		17 355 (5)		26 857 (5)
	15 years		39 144 (12)		58 271 (12)
	19 years		51 418 (16)		77 668 (16)
	20 years		105 292 (33)		163 232 (33)
Atrial fibrillation (incident)	Control	316 912	307 352 (97)	495 772	480 007 (97)
	Case		9560 (3)		15 765 (3)
Coronary artery disease (incident)	Control	317 055	302 574 (95)	481 533	458 689 (95)
	Case		14 481 (5)		22 844 (5)
Type 2 diabetes (incident)	Control	316 406	305 327 (96)	492 726	472 098 (96)
	Case		11 079 (4)		20 628 (4)
Stroke (incident)	Control	320 120	314 191 (98)	497 151	487 084 (98)
	Case		5929 (2)		10 067 (2)

For a *P*-value of ≤5 × 10^–8^, the PGSs explained between 0.06% (atrial fibrillation) and 14% (systolic blood pressure) of variance in the phenotypes (Supplementary Table S5, available as Supplementary data at *IJE* online).

### Association between educational attainment, polygenic scores and cardiovascular risk factors use

Educational attainment was associated with all cardiovascular risk factors/outcomes, except for LDL-C, although for all outcomes the effect was small (Supplementary Table S6, available as Supplementary data at *IJE* online). Except for alcohol consumption, higher educational attainment led to a reduction in the mean difference of all risk factors/outcomes (Supplementary Table S6, available as Supplementary data at *IJE* online).

### Effect modification by educational attainment of genetic susceptibility to cardiovascular risk factors

**Figure 1:**
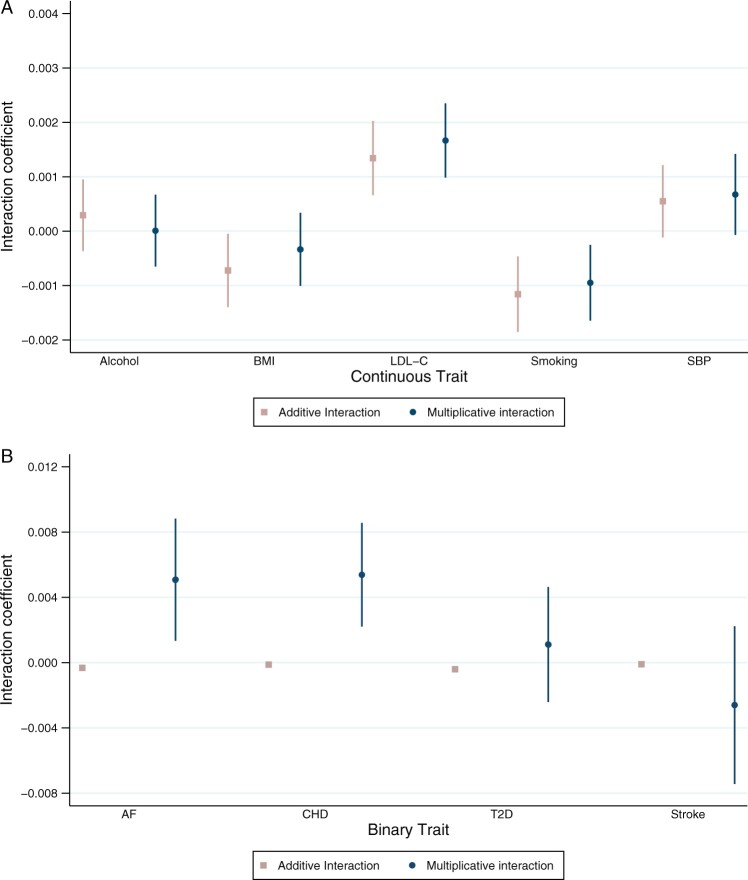
Coefficient for educational attainment as an effect modifier of polygenic susceptibility to cardiovascular risk factors or diseases on the additive and multiplicative scale. Analyses adjusted for age, sex and 40 genetic principal components. Alcohol = drinks per week; BMI = body mass index; LDL-C = low-density lipoprotein cholesterol; smoking = lifetime smoking behaviour; SBP = systolic blood pressure; AF = atrial fibrillation; CHD = coronary heart disease; T2D = type 2 diabetes. Analyses for binary outcomes on the multiplicative scale are presented as log odds ratios

**Figure 2:**
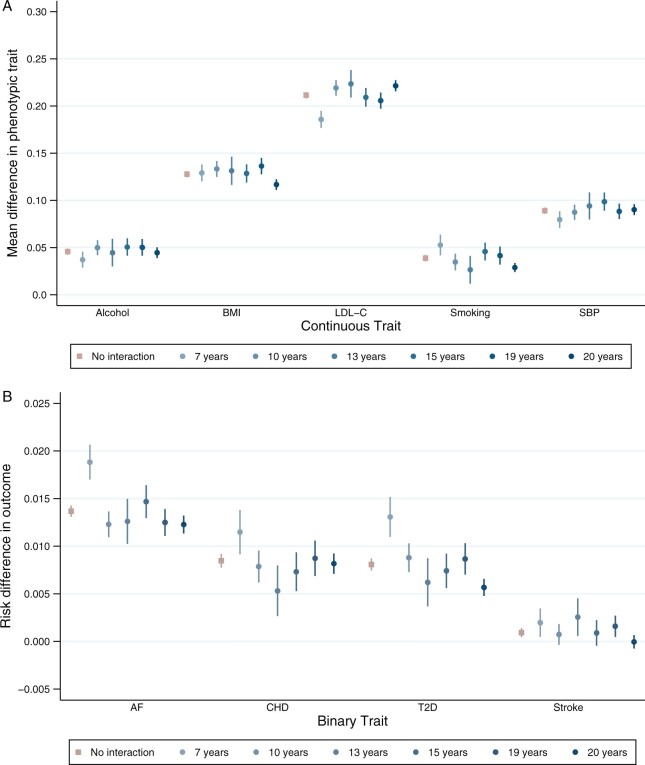
Association between polygenic scores for susceptibility to cardiovascular risk and phenotypic measure of each risk factor, stratified by educational attainment demonstrating effect modification on the additive scale. Analyses adjusted for age, sex and 40 genetic principal components. Alcohol (drinks per week) *P*_EM_ = 0.384; body mass index (BMI) *P*_EM_ = 0.036; low-density lipoprotein cholesterol (LDL-C) *P*_EM_ = 1.12 × 10^–4^; lifetime smoking behaviour *P*_EM_ = 0.001; systolic blood pressure (SBP) *P*_EM_ = 0.104; atrial fibrillation (AF) *P*_EM_ = 9.03 × 10^–8^; coronary heart disease (CHD) *P*_EM_ = 0.103; type 2 diabetes (T2D) *P*_EM_ = 3.23 × 10^–10^; stroke *P*_EM_ = 0.036. *P*_EM_ = *P*-value for effect modification

**Figure 3:**
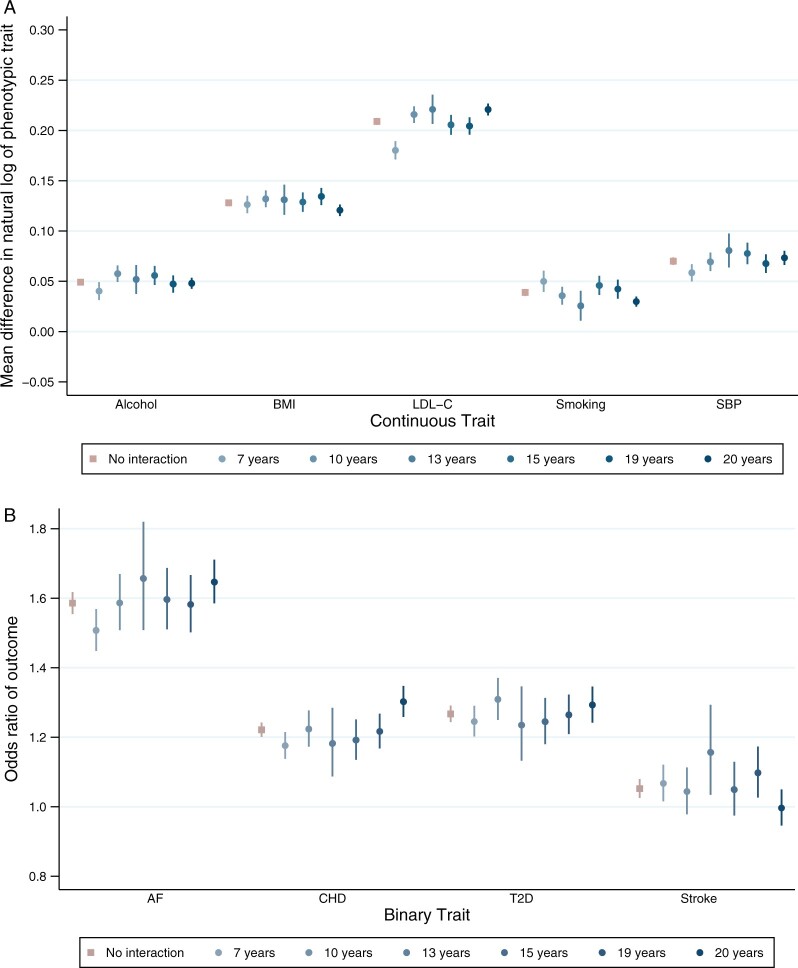
Association between polygenic scores for susceptibility to cardiovascular risk and phenotypic measure of each risk factor, stratified by educational attainment demonstrating effect modification on the multiplicative scale. Analyses adjusted for age, sex and 40 genetic principal components. Alcohol (drinks per week) *P*_EM_ = 0.976; body mass index (BMI) *P*_EM_ = 0.330; low-density lipoprotein cholesterol (LDL-C) *P*_EM_ = 1.63 × 10^–6^; lifetime smoking behaviour *P*_EM_ = 0.008; systolic blood pressure (SBP) *P*_EM_ = 0.076; atrial fibrillation (AF) *P*_EM_ = 0.008; coronary heart disease (CHD) *P*_EM_ = 8.94 × 10^–4^; type 2 diabetes (T2D) *P*_EM_ = 0.537; stroke *P*_EM_ = 0.292. *P*_EM_ = *P*-value for effect modification

For most PGSs, there was evidence that educational attainment modified the effect of the PGS on either the additive or multiplicative scale (Figures 1–3 and Supplementary Table S7 and S8, available as Supplementary data at *IJE* online). The exception was alcohol consumption, for which there was little evidence on either scale.

On the additive scale, higher educational attainment protected against genetic susceptibility to higher BMI, smoking, atrial fibrillation and type 2 diabetes (Figures 1 and 2). For example, a 1 SD increase in PGS for smoking increased the mean difference in lifetime smoking by 0.05 SD [95% confidence interval (CI): 0.04 to 0.06] for those with 7 years of education and by 0.03 SD (95% CI: 0.02 to 0.03) for people with 20 years of education (Figures 1 and 2 and Supplementary Table S7, available as Supplementary data at *IJE* online).

Also on the additive scale, higher educational attainment increased genetic susceptibility to LDL-C and systolic blood pressure. For example, for those with 7 years of education, an increase of 1 SD in the PGS for LDL-C increased mean LDL-C by 0.19 SD (95% CI: 0.18 to 0.19) compared with 0.22 SD (95% CI: 0.22 to 0.23) for people with 20 years of education per SD increase in PGS (Figures 1 and 2 and Supplementary Table S7, available as Supplementary data at *IJE* online).

On the multiplicative scale, there was evidence that higher educational attainment increased genetic susceptibility to atrial fibrillation and CHD. For example, for a 1 SD increase in atrial fibrillation PGS, the odds ratio for atrial fibrillation in individuals with 7 years of education was 1.59 (95% CI: 1.45 to 1.57) and for people with 20 years of educational attainment the odds ratio was 1.65 (95% CI: 1.59 to 1.71) (Figures 1 and 3 and Supplementary Table S8, available as Supplementary data at *IJE* online). There was little evidence of modification by education on the multiplicative scale for all other PGSs.

For all outcomes, the size of the coefficients for effect modification was small. Non-linear effects by strata of educational attainment were observed for a number of outcomes, including LDL-C, smoking, atrial fibrillation, CHD and type 2 diabetes. For some outcomes, such as with BMI, the effect modification is observed at a single level of educational attainment (Figures 2 and 3).

### Secondary analyses

Analyses using more liberal *P*-value thresholds for the PGS were broadly consistent with the main results. Similar directions of effect were observed, e.g. on the additive scale, a one-unit increase in educational attainment protected against genetic susceptibility to BMI and lifetime smoking behaviour (Supplementary Table S9 and S10, available as Supplementary data at *IJE* online).

## Discussion

In this analysis of UK Biobank, we found evidence that educational attainment modified the risk of genetic susceptibility to some, but not all, cardiovascular risk factors/outcomes. Our a priori hypothesis was that higher levels of education would mitigate genetic susceptibility to cardiovascular risk. However, in several cases, the effect modification was in the other direction, i.e. higher education accentuated genetic predisposition. Furthermore, the magnitude of the differences in associations between PGSs and cardiovascular risk factors/outcomes across levels of educational attainment was small in all cases. These results suggest that modification of the effect of PGSs by educational attainment is unlikely to play a substantial role in the generation of educational inequalities in CVD.

### Results in context

A number of studies have sought to identify the interplay between genetic susceptibility to cardiovascular risk factors with a range of lifestyle and environmental factors.^29–34^ However, few have considered the role of SEP interacting with genetic risk or investigated a wide range of cardiovascular risk factors/outcomes.

Two recent studies using UK Biobank demonstrated that a greater Townsend deprivation index score accentuated the genetic risk of obesity.^8^^,^^9^ In contrast to our results, the previous literature has not found evidence that education modifies the genetic risk of obesity.^9^^,^^10^ This may be related to power, where previous studies have used smaller sample sizes to estimate interactions.

These differences could also be due to the education definition used. Here, we used the ISCED years of schooling measure, whereas previous research has used age of completing full-time education^9^ and highest qualification.^10^

Typically, non-linear associations were observed when stratifying by years of education, demonstrating that years of education is not a homogenous exposure. For many outcomes, including LDL-C, smoking, atrial fibrillation, CHD and type 2 diabetes, effect modification was driven by individuals with the lowest levels of education. These non-linear effects may be explained by later measures of adult SEP. Much of the variation in educational attainment is determined by early adulthood and therefore does not capture later-life factors that may be important in the development of cardiovascular inequalities, such as occupation or income.

### Strengths and weaknesses, and caveats to the analysis of effect modification

There are a number of strengths in this study. Many previous analyses of gene*environment interactions in CVD rely on candidate gene studies,^33^^,^^35^^,^^36^ often resulting in spurious associations.^37^ We have used PGSs for nine cardiovascular risk factors/outcomes. Whilst candidate gene studies focus on a single genetic variant, or a small group of (common) genetic variants that individually explain a large(r) amount of the variance in the trait, PGSs include a large number of genetic variants, each explaining a small amount of the variation, but cumulatively explaining a large amount.^38^^,^^39^ For most diseases, including CVD, polygenic inheritance of these common variants plays a greater role than rare monogenic mutations.^39^^,^^40^ Therefore, the broad measure of genetic susceptibility used here is likely to represent a greater number of biological pathways for the aetiology of CVD.

We created PGSs at a range of *P*-value thresholds. At a more stringent threshold (e.g. *P* ≤ 5 × 10^–8^), the genetic variants included are less likely to be pleiotropic (i.e. also associated with different phenotypes), but the variance explained by the PGS may be lower than with a more liberal threshold (e.g. *P* ≤ 0.5). Additionally, less-stringent clumping thresholds were used to improve polygenic prediction, but this may introduce pleiotropic SNPs. However, sample overlap was present in the atrial fibrillation summary statistics used to derive the PGS, where UK Biobank contributed 60% to the GWAS, which may lead to overestimated effect sizes. However, sensitivity analyses using non-overlapping samples were consistent.

The lack of evidence for effect modification between education and the PGS for alcohol consumption observed here could be due to insufficient power to detect an interaction or because of the variable definition. Alcohol consumption was defined as drinks per week, but the type of alcoholic drink consumed may be an important factor. Additionally, alcohol consumption was self-reported by participants, which is prone to recall bias.^41^^,^^42^ If this recall bias is differential by educational attainment, this may mask any effect modification between educational attainment and genetic susceptibility to alcohol consumption. Alternatively, different patterns of drinking may occur by strata of educational attainment. It has been shown that individuals of lower SEP are more likely to drink to extreme levels,^43^ but individuals of higher SEP consume similar or even greater amounts of alcohol.^44^

Where effect modification was found, should different definitions of the outcome variables be used, e.g. smoking initiation as opposed to lifetime smoking behaviour, the observed evidence of effect modification may change. Similarly, the effect modification identified here may differ for alternative measures of SEP.

These results may be specific to the model used to derive PGSs. For example, a recent GWAS of systolic blood pressure demonstrated that educational attainment interacts with the genetic architecture of blood pressure.^45^ If these interactions were accounted for when deriving the PGS, different results may be observed.

Low statistical power reduces the chance of detecting a true effect should one exist.^46^ Although some power calculators have been developed to calculate power in gene*environment interaction analyses,^47^ to our knowledge, none has been developed for use with PGSs. Additionally, power calculations rely on making assumptions about the true effect size, which is difficult to estimate in this case. Therefore, we believe it is more informative to interpret the results and likely power based on the point estimates, standard errors and width of the confidence intervals. However, it is possible that we did not detect effect modification by education on the effects of alcohol consumption due to insufficient power to detect an interaction, as demonstrated by small point estimates and wide confidence intervals.

Studies of effect modification can be biased by reverse causality and confounding. Analyses of CVD outcomes were restricted to incident cases to avoid bias from behaviour changes following a CVD diagnosis to avoid reverse causality. Genetic variants are determined at conception and therefore not affected by unmeasured later-life confounding factors. However, they can be confounded by population structure.^48^ In this analysis, we controlled for genetic principal components to minimize this bias.

It has been suggested that further to controlling for confounders, the interaction between the (i) confounders and environmental exposure and (ii) confounders and genetic exposure should be controlled for.^49^^,^^50^ This avoids specification error by accounting for the covariation between the confounders and the interactions tested. However, due to the large number of principal components included as confounders in these analyses, there is not enough variation in the data to include these additional covariates. Therefore, these analyses may be biased by residual confounding.

UK Biobank participants are typically more highly educated and of a higher SEP than the UK population.^14^ Therefore, evidence of effect modification by education in this sample may be due to collider bias caused by non-random selection into the study.^14^^,^^51^

These results do not specifically identify what it is about educational attainment that modifies genetic susceptibility to cardiovascular risk. For example, remaining in education may lead to an increased knowledge of the smoking harms, even if they have genetic variants increasing their susceptibility to heavier smoking.^52^ Indeed, a number of alternative factors associated with educational attainment could be contributing to the effect modification. For example, parental genotype or family-level environmental factors may explain both educational attainment and differential effects of PGSs by strata of education.

Due to limited power, we have not used causal inference methods to test whether education is causal with respect to all outcomes. To understand whether effect modification by education is causal, exogenous exposures for education, such as the Raising of the School Leaving Age (RoSLA), could be used. However, it is challenging to identify sufficiently large samples that were both exposed to the RoSLA and have genotypic data.

Where educational attainment increased genetic susceptibility to CVD, such as for atrial fibrillation, effect modification may be due to differential rates of diagnosis, which may independently contribute to cardiovascular inequalities. Whilst risk factors such as BMI and smoking were measured near universally in participants at baseline, CVD was ascertained through linkage to hospital inpatient records.

### Interpreting analyses of interaction and effect modification

The terms interaction and effect modification are often used interchangeably in modern epidemiology. Whilst statistically the same, the distinction can be made where an interaction is defined in terms of the effects of two causal risk factors, whereas effect modification specifies that the effect of one risk factor varies by strata of a second factor, the effect of which on the outcome is not necessarily causal.^53^ We have used the term ‘effect modification’ throughout this analysis, where we specifically hypothesize that the effect of the PGSs varies by strata of educational attainment. This term also acknowledges that we have not explicitly tested the causal associations between (i) educational attainment and (ii) PGSs on each outcome.

Interaction and effect modification have often been dichotomized into ‘biologic interaction’ and ‘statistical interaction’.^54^^,^^55^ Biologic interaction is said to be a deviation from an additive effect of two risk factors on the risk difference of the outcome. However, this term has been criticized for being difficult to interpret and giving potentially misleading assurances about causal biological mechanisms that have not been assessed.^55^

Statistical interaction is described as the deviation from the expected effect of two joint risk factors, under the assumption the risk factors are independent on the additive or the multiplicative scale.^54^ When two risk factors are associated with the outcome, there should always be evidence of an interaction on at least one scale, so we present results on both the additive and the multiplicative scales.^13^ This is an important distinction from previous analyses, which have typically only reported results on the additive scale.^8^^,^^9^ A full discussion of additive and multiplicative interactions can be found elsewhere.^13^

### Public health relevance

To determine the public health relevance of these results, it is important to interpret the magnitude and direction of any effect modification. Coefficients for effect modification were uniformly small in this analysis and the direction of the effect across outcomes was not consistent. This indicates that any effect modification by educational attainment on the effect of genetic susceptibility to cardiovascular risk factors/outcomes is unlikely to contribute to the development of inequalities in cardiovascular risk. Although some results were scale-dependent—e.g. greater evidence of effect modification by education on genetic susceptibility to type 2 diabetes on the additive scale—the direction of effect modification was generally consistent within outcomes across both the additive and multiplicative scales. Given the small coefficients for effect modification, these differences in precision are likely driven by low power.

## Conclusions

In this study, we found that educational attainment modifies genetic susceptibility to a number of cardiovascular risk factors/outcomes. The direction of this effect was mixed and the size of the effect-modification coefficients was small. This suggests that effect modification by educational attainment on the effect of genetic susceptibility to cardiovascular risk factors/outcomes is unlikely to explain the development of inequalities in cardiovascular risk.

## Ethics approval

This research was conducted using the UK Biobank resource using the approved application 10953.

## Author contributions

A.R.C. designed the study, cleaned and analysed the data, interpreted the results, and wrote and revised the manuscript. S.H. assisted with data analysis, interpreted the results and critically reviewed and revised the manuscript. D.G. interpreted the results and critically reviewed and revised the manuscript. G.D.S., A.E.T., N.M.D. and L.D.H. all designed the study, interpreted the results, critically reviewed and revised the manuscript, and provided supervision for the project. N.M.D. and L.D.H. contributed equally and are joint senior authors of this manuscript. A.R.C. and N.M.D. serve as guarantors of the paper. The corresponding author attests that all listed authors meet authorship criteria and that no others meeting the criteria have been omitted.

A.R.C. affirms that the manuscript is an honest, accurate and transparent account of the study being reported and no important aspects of the study have been omitted.

## Supplementary Material

dyac002_Supplementary_Data

## Data Availability

The data used in this study have been archived with the UK Biobank study. Summary data for external weightings of polygenic scores are available from each GWAS. The code used to derive polygenic scores is available at https://github.com/sean-harrison-bristol/UK_Biobank_PRS/blob/master/mrbase_grs_v3.01.R and the analysis code is available at github.com/alicerosecarter/gxe_cv_riskfactors.
