# Correction to: Educational attainment as a modifier for the effect of polygenic scores for cardiovascular risk factors: cross-sectional and prospective analysis of UK Biobank

**DOI:** 10.1093/ije/dyac169

**Published:** 2022-08-22

**Authors:** Alice R Carter, Sean Harrison, Dipender Gill, George Davey Smith, Amy E Taylor, Laura D Howe, Neil M Davies

First published online 3 February 2022, *International Journal of Epidemiology*, https://doi.org/10.1093/ije/dyac002

The summary statistics used to derive the atrial fibrillation polygenic score were mistakenly attributed to Ellinor (2012) genome-wide association study, but were actually those from Roselli (2018). This error did not affect conclusions of the paper.

The paper and supplementary material have been corrected online.

